# Pulmonary Sequestration Mimicking Symptoms of Ischemic Heart Disease

**DOI:** 10.7759/cureus.40200

**Published:** 2023-06-09

**Authors:** Aatma Ram, FNU Warsha, FNU Vinisha, Dilpat Kumar, Muskan Kumari

**Affiliations:** 1 Hospice and Palliative Medicine, Lehigh Valley Health Network, Allentown, USA; 2 Internal Medicine, Rosalind Franklin Medical University, McHenry, USA; 3 Internal Medicine, Interfaith Medical Center, New York, USA; 4 Internal Medicine, Liaquat National Hospital, Karachi, PAK; 5 Cardiology, East Tennessee State University, Johnson City, USA; 6 General Medicine, Liaquat University of Medical and Health Sciences, Jamshoro, PAK

**Keywords:** rare lung disease, intralobar pulmonary sequestration, recurrent pneumonia, congenital pulmomonary abnormalities, broncho-pulmonary sequestration

## Abstract

Pulmonary sequestration is a rare congenital abnormality characterized by the presence of a nonfunctional lobe of the lung being separated in both blood flow and function from the rest of the lung. The condition may go unrecognized on prenatal imaging and present during adolescence and young adulthood with cough, chest pain, shortness of breath, and recurrent pneumonia. However, some patients may remain asymptomatic until later adulthood and be diagnosed based on incidental imaging findings. Surgical resection is the recommended treatment for this condition, although controversy exists regarding its use in asymptomatic patients and adults. In this case report, we present a case of a 66-year-old man who presented with progressively worsening dyspnea on exertion and atypical chest pain and underwent an ischemic workup to rule out coronary artery disease. The extensive diagnostic evaluation led to the diagnosis of nonobstructive coronary artery disease and left-sided pulmonary sequestration. The patient subsequently underwent surgical resection of the left lower pulmonary lobe, resulting in a significant improvement in symptoms.

## Introduction

Pulmonary sequestration (PS) is a rare, congenital pulmonary anomaly characterized by a segment or lobe of dysplastic, nonfunctional lung tissue that lacks communication with the tracheobronchial tree and receives an anomalous systemic vascular supply, separated from the rest of the lung [1,2]. There are two types. Intralobar sequestration (ILS) refers to a condition where abnormal lung tissue shares its visceral pleural lining with the surrounding normal lung tissue. On the other hand, extralobar sequestration (ELS) is a condition where the abnormal lung tissue is separate from the surrounding lung tissue and has its own visceral pleura. Pulmonary sequestration accounts for 0.15% to 6.4% of congenital pulmonary malformations [2]. While most cases can be detected on prenatal imaging or present during the first decade of life, PS can rarely remain undiagnosed until late adulthood. Pulmonary sequestration presentation is nonspecific for cardiopulmonary illness resulting in a delayed diagnosis and patient morbidity. The commonest symptoms are cough, chest pain, hemoptysis, shortness of breath, and recurrent pneumonia [2-4].

## Case presentation

A 66-year-old man with a medical history of hypertension, obstructive sleep apnea (OSA), postnasal discharge, and recurrent pneumonia presented to our clinic with a progressively worsening productive cough, exertional dyspnea, and atypical chest pain for the past five years. He denied fever, chills, sore throat, weight loss, palpitations, orthopnea, and nocturnal dyspnea. The physical examination revealed an obese elderly man with an elevated neck circumference of 18 cm and mild bilateral nasal turbinate hypertrophy. Vital signs were within normal limits, and the cardiopulmonary examination was unremarkable. Initial lab work, including a complete blood count (CBC), comprehensive metabolic panel (CMP), and electrocardiogram, was unremarkable. The patient underwent a computed tomography (CT) cardiac calcium score protocol to assess for coronary artery disease (CAD) at cardiology recommendations, which revealed a high calcium score of 264 in the left anterior descending artery (LAD), suggestive of moderate plaque burden, as well as bronchial wall thickening with airspace disease in the left lower lobe, concerning for atelectasis or pneumonia. A transthoracic echocardiogram showed a normal left ventricular ejection fraction and no significant valvular abnormality.

**Figure 1:**
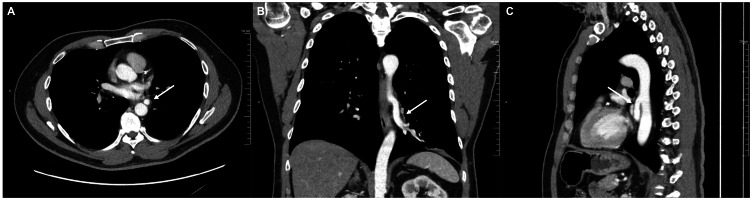
CT chest with contrast illustrating the finding of an anomalous left pulmonary artery supplying the left lower lobe arising from the descending thoracic aorta, indicated by the arrow in (A) transverse, (B) coronal, and (C) sagittal views. CT: computed tomography.

A CT chest with contrast was obtained, which showed loss of lung volume in the inferior aspect of the left lower lobe with increased hazy opacities, and an anomalous left pulmonary artery, raising suspicion for pulmonary sequestration (Figures 1A-1C).

**Figure 2:**
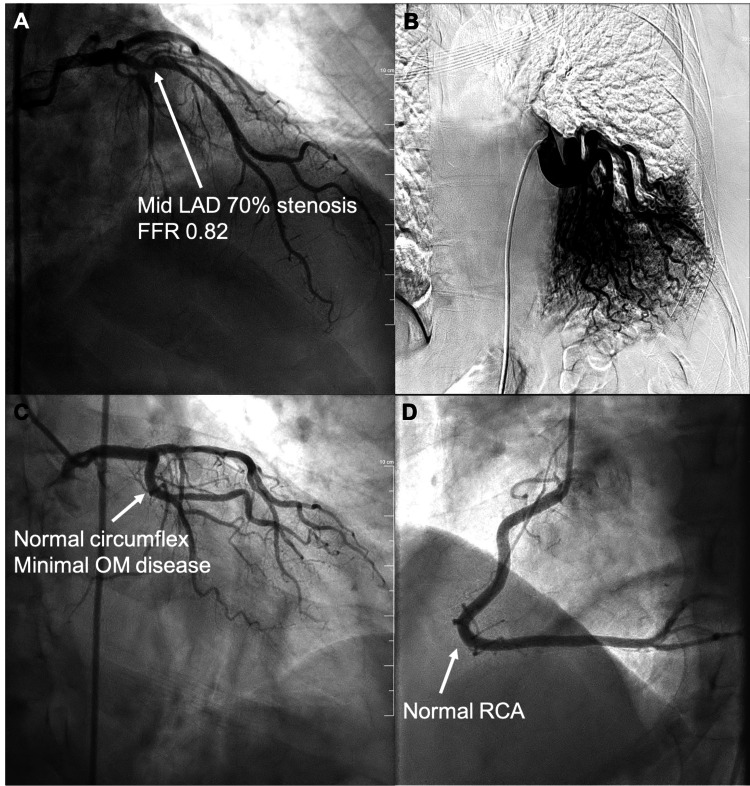
Right and left cardiac catheterization with aortogram images. (A) 70% stenosis of the left anterior descending artery with a flow fractional reserve of 82%. (B) Distinct blood flow to the lower lobe of the left lung arising from the thoracic aorta. (C) Normal circumflex branch of the left coronary artery with minimal disease in the obtuse marginal branches. (D) Normal right coronary artery. LAD: left anterior descending artery.

Further workup was pursued to evaluate for cardiac disease, and the patient underwent left- and right-sided cardiac catheterization with an aortogram. The results showed 70% occlusion of the mid-left anterior descending artery (Figure 2A), and confirmed interlobar pulmonary sequestration with the origin of the interlobar bronchial/pulmonary artery from the descending thoracic aorta, supplying the left lower lobe of the lung.

Based on these findings and clinical history, the patient was referred to cardiothoracic surgery and underwent surgical resection of the left lower pulmonary lobe. His coronary artery disease was managed medically. Two months after the procedure, he had a significant improvement in his symptoms of dyspnea and continued to follow up with his cardiothoracic surgeon as an outpatient.

## Discussion

Pryce (1964) introduced the term sequestration, which is derived from the Latin word “sequestrare,” meaning “to separate” [5]. Pulmonary sequestration (PS) acts as a separate, nonfunctional lung unit that lacks normal communication with the tracheobronchial tree. It differs from normal lung tissue because it receives oxygenated blood rather than nonoxygenated blood. In most cases, it receives blood supply from the thoracic aorta (74%), while in the remainder of cases, blood supply originates from the abdominal aorta and its branches, including the gastric or splenic arteries [3,6]. Pulmonary sequestration is classified as intralobar and extralobar [3,4].

Intralobar sequestration accounts for 80% of cases [4,7] and is characterized by a lesion that is located within the normal lobe and does not have its own visceral pleura, while an extralobar lesion is located outside the lung and has its own visceral pleura [3,4,6,7]. Most intralobar sequestrations drain into pulmonary veins, whereas extralobar sequestrations drain into azygous and hemi-azygous veins or inferior vena cava [5]. For an unknown reason, these sequestrations are predominantly found on the left side accounting for two-thirds of cases [8].

Most of the patients with PS (~50%) remain asymptomatic [5]. Cough is the most common presenting symptom reported in 34% of cases, followed by dyspnea, thoracic pain, and hemoptysis [5,9]. Recurrent respiratory infections were reported in 16% of the patients [5]. Our patient presented with nonspecific symptoms, and the diagnosis was challenging due to the presence of comorbid coronary artery disease.

The pathogenesis of pulmonary sequestration is not fully understood. However, the most widely accepted theory is that it results from the formation of an accessory lung bud caudal to the normal lung buds during the early embryonic period [9]. In ILS, accessory lung buds develop before the formation of the pleura; the same pleura thus covers both the normal and sequestered lung tissue. Whereas extralobar sequestration develops as an accessory lung bud after the formation of the pleura, and the sequestered lung tissue forms its own pleural covering, resulting in ELS.

Prenatal ultrasonography plays an important role in early diagnosis [10,11]. Computed tomography (CT) scan with intravenous contrast and preferably CT angiography (CTA) is the method of choice for identifying PS in children and adults. PS can manifest with multiple radiologic findings on computed tomography (CT) that resemble a mass or a consolidation with or without cysts, bronchiectasis, and cavitary lesions. A retrospective review of imaging findings in 32 patients by Alsumrain et al. revealed common radiological findings to be mass/consolidation in 61%, followed by hyper lucency in 42%; cystic changes were noted in 23% of cases. Dilated bronchi were seen in 15%, and mixed radiologic features were found in 34% of the patients [5].

Considering the rarity of the condition, the treatment strategies are based on expert opinions and limited available data retrieved from case reports, case series, and retrospective reviews. The surgical resection of the symptomatic pulmonary sequestrations remains the mainstay of treatment. Surgical recommendations are unclear in asymptomatic patients; however, some experts favor surgical resection of PS due to concerns regarding infectious complications. However, this issue remains debatable since data regarding the long-term clinical course and outcome of those with unresected PS are sparse, particularly in the adult population. Our patient underwent surgical evaluation at a tertiary center and opted to undergo surgical resection. We recommend weighing the benefits of surgical resection against the procedural and post-procedural complications.

## Conclusions

Pulmonary sequestration syndrome is a rare congenital anomaly where part of the lung is separated in both blood flow and function and is usually a nonfunctional lobe. While most cases can be diagnosed through prenatal screening and during childhood, rare cases may remain asymptomatic and undiagnosed until late adulthood. Clinical presentation is nonspecific for a cardiopulmonary illness resulting in a lack of workup and delayed diagnosis. The recommended treatment for symptomatic pulmonary sequestration is surgical resection; however, the management of incidentally detected cases in asymptomatic patients remains debatable. This case highlights the intersection and interconnectedness between pulmonary and cardiac disease and adds to the available literature in this regard for future research. 
